# Supplementary material for: Protective Effect of Low 2-O, 3-O Desulfated Heparin (ODSH) Against LPS-Induced Acute Lung Injury in Mice
Source: Biomolecules. 2025 Aug 26;15(9):1232. doi: 10.3390/biom15091232 (PMC12467875; doi:10.3390/biom15091232)
Supplement: Supplementary file 1 [file biomolecules-15-01232-s001.zip › biomolecules-3761226-supplementary.pdf]

Table S1: Primer sequences used for RT-qPCR analysis

Gene name	Forward sequence (5'→3')	Reverse sequence (5'→3')	OriGene ID
p38	CCGAACGATACCAGAACCTGTC	ACGCAACTCTCGGTAGGTCCTT	NM 011951
NF-κB	TCCTGTTCGAGTCTCCATGCAG	GGTCTCATAGGTCCTTTTGCGC	NM 009045
IL-6	TACCACTTCACAAGTCGGAGGC	TACCACTTCACAAGTCGGAGGC	NM 031168
GAPDH	CATCACTGCCACCCAGAAGACTG	ATGCCAGTGAGCTTCCCGTTCAG	NM 008084

All the primers used for mouse RT-qPCR analysis were referenced from OriGene Technologies, Inc. (USA) and synthesized by Integrated DNA Technologies (IDT, Coralville, IA, USA).

Amplification conditions: Stage 1: 50°C for 2 min, 95°C for 2 min; Stage 2: 40 cycles (95°C for 15 sec, 60°C for 15 sec and 72°C for 1 min).
Dissociation curve conditions (melt curve stage): 95°C for 15 sec, 60°C for 1 min and 95°C for 15 sec.

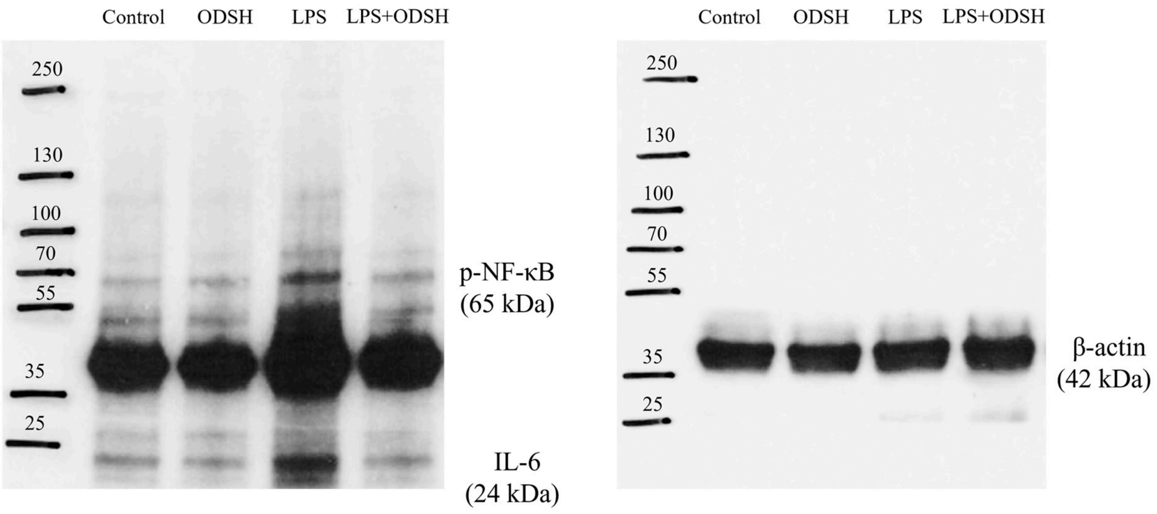

Figure S1: Western blot images illustrate the expression levels for p-NF-κB and IL-6. β-actin served as a loading control to verify equal protein quantities across all samples. In each group 15 μg of protein sample was loaded, all drawn from the same aliquot to ensure uniformity between test and control groups.

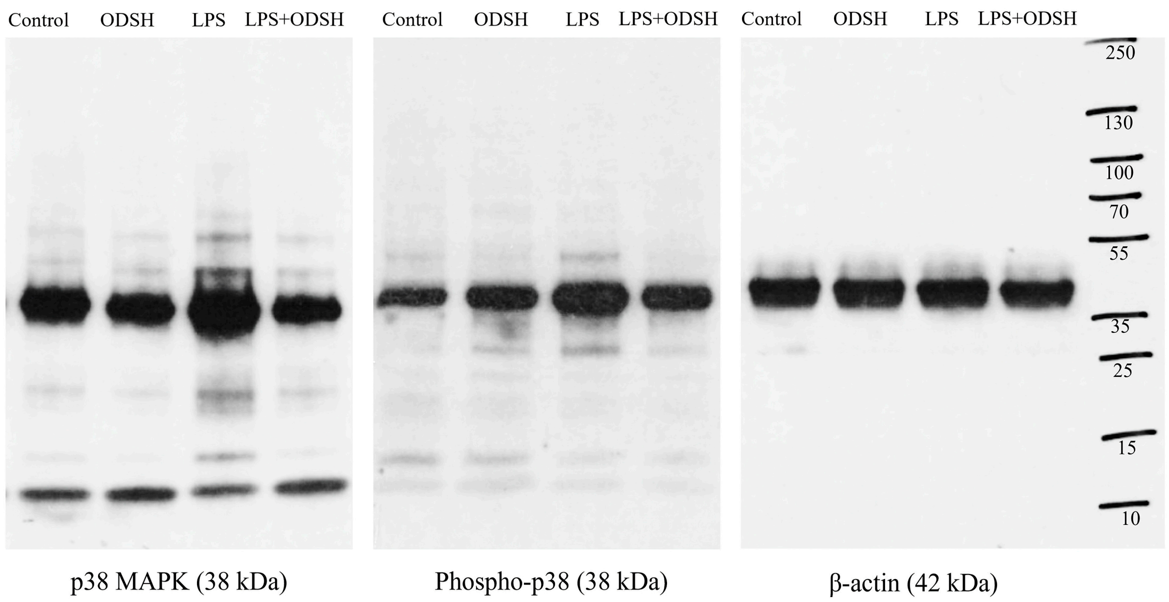

Figure S2: Western blot analysis of p38 MAPK and phospho-p38 protein levels. β-actin was used as an internal loading control to ensure equal protein loading across samples. For each experimental group, 15 µg of protein sample was loaded from the same aliquot to maintain consistency between test and control conditions.
